# The effects of social story interventions on individuals with Autism Spectrum Disorders and influencing factors: a meta-analysis of single-case experimental studies

**DOI:** 10.3389/fpsyg.2026.1855311

**Published:** 2026-06-25

**Authors:** Tianwei Guo, Yuhao Wu, Meng Xia, Huiyuan Chen, Qian Shao, Fangyuan Peng, Jianxing Chen

**Affiliations:** 1 School of Teacher Education, Chifeng University, Chifeng, China; 2 School of Educational Science, Chifeng University, Chifeng, China; 3 School of Stomatology, Chifeng University, Chifeng, China

**Keywords:** Autism Spectrum Disorder, influencing factors, meta-analysis, SCEDs, Social Story interventions

## Abstract

**Purpose:**

This study aimed to evaluate the overall effectiveness of Social Story interventions for individuals with Autism Spectrum Disorder (ASD) and to examine the moderating effects of key participant and intervention characteristics.

**Method:**

A meta-analysis of 21 single-case experimental design studies, encompassing data from 61 participants with ASD, was conducted. The overall effect size and moderator analyses were calculated using the Tau-U index.

**Result:**

The aggregated analysis revealed a moderate overall effect (Tau-U = 0.743). Moderator analyses indicated that the intervention was most effective for school-aged children (7–12 years). Significant positive effects were found for improving social skills, safety skills, and reducing problem behaviors, but not for classroom adaptive skills. While digital formats yielded a slightly higher effect size than paper-based versions, this difference was not statistically significant. The intervention effect was not significantly moderated by the implementer's identity (therapist, teacher, parent, or researcher) or participant gender.

**Conclusion:**

Social Story interventions are an efficacious approach with moderate overall effects for individuals with ASD, particularly school-aged children and for specific skill domains. The findings support the flexibility of implementation across various agents. Future research should expand sample diversity, investigate the integration of emerging technologies, and focus on enhancing the long-term maintenance and generalization of outcomes.

## 1 Introduction

### 1.1 Autism Spectrum Disorder and intervention needs

Autism Spectrum Disorder (ASD) constitutes a group of complex neurodevelopmental conditions primarily characterized by persistent deficits in social communication and social interaction, alongside restricted, repetitive patterns of behavior, interests, or activities (Sharma et al., 2018). Current prevalence estimates indicate that ASD affects approximately 1 in 36 children, with a well-documented male-to-female ratio of around 4:1 (Sharma et al., 2018; Genovese and Butler, 2023). However, growing evidence suggests this gender disparity may partly reflect differences in phenotypic presentation and diagnostic bias, as females often exhibit subtler symptoms or develop sophisticated “camouflaging” strategies, leading to under-identification or misdiagnosis (Green et al., 2019; Ochoa-Lubinoff et al., 2023). The etiology of ASD involves a significant genetic component, with heritability estimates between 70% and 90%, and involves complex interactions among hundreds of genes, chromosomal variations, and environmental factors influencing neurodevelopment (Genovese and Butler, 2023).

A hallmark of ASD is its profound heterogeneity, manifesting not only in the wide spectrum of symptom severity and intellectual ability but also in the high prevalence of co-occurring psychiatric and medical conditions. Common comorbidities include Attention-Deficit/Hyperactivity Disorder (ADHD), anxiety, depression, and gastrointestinal issues (Sharma et al., 2018; Antshel and Russo, 2019). This heterogeneity underscores the critical need for highly individualized and evidence-based intervention approaches tailored to each individual's unique profile (Aishworiya et al., 2024). The core social communication deficits often translate into significant challenges in understanding social cues, interpreting others' perspectives, and engaging in reciprocal interaction. Furthermore, executive function impairments and difficulties with behavior regulation are frequent, impacting safety awareness, adaptive functioning, and classroom participation (Antshel and Russo, 2019).

While behavioral interventions remain the cornerstone of treatment for core ASD symptoms, the search for effective supports is ongoing and multifaceted (Aishworiya et al., 2024). Established approaches like Applied Behavior Analysis (ABA) are often complemented by specialized therapies targeting social skills, communication, and sensory integration. Pharmacological treatments are frequently utilized to manage co-occurring conditions such as ADHD, anxiety, or irritability, though they do not address core ASD deficits directly (Sharma et al., 2018; Aishworiya et al., 2024). Emerging areas of research highlight the potential role of the gut-brain axis and novel psychopharmacological targets, pointing to future avenues for intervention (Aishworiya et al., 2024; Wang et al., 2020; Sharon et al., 2019; Zhao et al., 2020). Given the complexity and lifelong nature of ASD, developing, evaluating, and refining accessible, effective, and personalized interventions that address both core symptoms and functional impairments remains a paramount priority in the field (Dawson, 2008).

### 1.2 Social story intervention: concept and theoretical foundation

Among the diverse behavioral interventions for ASD, Social Stories™ stand out as a widely adopted, narrative-based approach. Originally developed by Carol Gray in 1991, a Social Story is defined as “a short story—defined by specific criteria—that describes a situation, concept, or social skill using a format that is meaningful for people with ASD” (Wright et al., 2016). The primary goal is to share accurate, easily understood social information to clarify social contexts, explain perspectives, and outline expected behaviors, thereby reducing anxiety and confusion in specific situations (Wright et al., 2016; Wright et al., 2014).

A key feature of Social Stories is their structured composition, typically incorporating four core types of sentences according to Gray's guidelines: “Descriptive” sentences (objectively stating the where, who, and what of a situation), “Perspective” sentences (describing the internal states, thoughts, or feelings of others), “Directive” sentences (gently suggesting a desired behavioral response), and “Affirmative” sentences (expressing a shared value or rule) (Wright et al., 2016). This structured narrative aims to make abstract social expectations concrete and predictable.

The theoretical rationale for Social Stories is multifaceted, drawing from several frameworks central to understanding ASD. First, they are grounded in addressing impairments in “Theory of Mind (ToM)”—the ability to infer the mental states of others. By explicitly stating others' perspectives and feelings, Social Stories aim to scaffold this understanding (Amorim et al., 2025; Bayliss et al., 2019; Scheeren et al., 2013). Second, they leverage “visual supports”, a well-established strategy for individuals with ASD who often demonstrate relative strengths in visual processing compared to auditory processing. The written text, often paired with pictures, provides a static, predictable reference that can be reviewed repeatedly (Hutchins and Prelock, 2018; Zorzi et al., 2025). Furthermore, Social Stories align with principles of “social learning theory” by modeling appropriate behaviors and outcomes within a narrative framework. Contemporary perspectives also suggest Social Stories may function by providing clear, rule-based (“if-then”) structures for navigating social situations, potentially aligning with a systemizing cognitive style proposed to be a relative strength in autism (Camilleri et al., 2023).

Traditionally, Social Stories have been delivered in “paper-based formats” (booklets or sheets), but evolving technology has fostered the development of “digital versions”. These can include interactive elements, audio narration, and animated features, potentially increasing engagement and accessibility, especially for younger children or those with co-occurring reading difficulties (Zorzi et al., 2025). The format can be tailored for individual or small-group use and implemented across settings (home, school, clinic) by various agents, including parents, teachers, or therapists (Wright et al., 2016; Wright et al., 2014).

In summary, Social Stories represent a manualized, theoretically informed intervention designed to translate complex social scenarios into comprehensible, rule-governed narratives. Their adaptability in format, content, and implementation context makes them a practical tool within the broader spectrum of social-behavioral interventions for ASD.

### 1.3 Empirical evidence and limitations of existing research

The empirical literature on Social Story interventions presents a complex and somewhat inconsistent picture, primarily comprising single-case experimental designs (SCEDs) with a growing number of group-based studies in recent years. Research to date has demonstrated that Social Stories can be effective in teaching a variety of skills and reducing challenging behaviors for some individuals with ASD. Positive outcomes have been documented in domains such as improving “oral hygiene and toothbrushing routines” (Wright et al., 2016; Zhou et al., 2020a,b), increasing on-task behavior and verbal initiations (Anthony and Bobzien, 2022), teaching “community living and safety skills” (Zorzi et al., 2025), and alleviating specific challenging behaviors (Rhodes, 2014). Systematic reviews often conclude a “largely positive picture” of effectiveness (Wright et al., 2016; Zhou N. et al., 2024).

However, findings are not uniformly positive or consistent. Inconsistent results are evident across different “outcome domains” and participant groups. While some studies report significant improvements in targeted, specific behaviors (e.g., steps in a toothbrushing routine), others find minimal or no effect on broader, more generalized measures of “social responsiveness or emotional health” (Wright et al., 2024, 2020). For instance, a large cluster randomized controlled trial (RCT) found Social Stories had no clinically significant impact on overall teacher-rated social responsiveness at 6 months, despite helping children meet specific individualized goals (Wright et al., 2024). Furthermore, the relative efficacy compared to other interventions, such as video modeling, remains unclear and may vary by target skill (Piraneh et al., 2023).

Several key “methodological limitations” constrain the current evidence base and contribute to these inconsistencies. First, the literature is dominated by “single-case designs” with very small sample sizes, limiting statistical power and generalizability (Zhou N. et al., 2024; Kokina and Kern, 2010; Goltstein et al., 2021). Second, there is a notable scarcity of high-quality, fully powered “randomized controlled trials” (RCTs) needed to establish robust causal evidence of effectiveness and cost-effectiveness (Zhou k. et al., 2024; Wright et al., 2024, 2020). Third, significant variability in “intervention integrity” is frequently reported; many studies do not adhere strictly to Gray's guidelines for Social Story construction or fail to report fidelity of implementation, making it difficult to discern the “active ingredients” of the intervention (Zhou k. et al., 2024; Kokina and Kern, 2010; Camilleri et al., 2022). Fourth, most studies have not systematically investigated how intervention effects are moderated by participant characteristics (e.g., age, cognitive profile, language ability) or intervention parameters (e.g., format, implementer, duration) (Kokina and Kern, 2010). While emerging research suggests factors like “digital delivery” may influence outcomes and user preference (Anthony and Bobzien, 2022; Camilleri et al., 2022, 2024), comprehensive moderator analyses are lacking. Finally, there is a paucity of research exploring the “long-term maintenance” of skills and the “generalization” of behaviors across settings (Kokina and Kern, 2010).

In summary, while Social Stories show promise as a practical intervention, the existing research is characterized by methodological heterogeneity, a reliance on small-N designs, and an incomplete understanding of the factors that influence their effectiveness. This underscores the need for a quantitative synthesis that can aggregate findings across single-case studies while systematically examining potential moderators to clarify for whom and under what conditions Social Stories are most effective.

### 1.4 Rationale and objectives of the current meta-analysis

Given the methodological limitations and inconsistent findings highlighted in the existing literature, a quantitative synthesis of the evidence on Social Story interventions is both timely and necessary. While narrative reviews and some meta-analyses exist (Baek and Luo, 2023), there remains a critical need for a comprehensive meta-analysis that specifically aggregates data from SCEDs, which constitute the majority of the evidence base (García-González et al., 2021; Moeyaert et al., 2023). Conducting a meta-analysis of SCEDs allows for the statistical aggregation of effects across studies, providing a more robust estimate of overall intervention effectiveness than narrative synthesis alone (Baek et al., 2023; Van den Noortgate and Onghena, 2024). Furthermore, such an approach enables the systematic investigation of potential moderating variables—such as participant characteristics and intervention parameters—that may explain the variability in outcomes observed across individual studies (Kang et al., 2025; Lim et al., 2023). This aligns with the broader shift toward identifying “what works for whom” in personalized intervention science (Goltstein et al., 2021; Maric et al., 2023).

To accurately quantify intervention effects from SCED data, an appropriate effect size metric is required. For this meta-analysis, we selected the “Tau-U index” (Lee and Cherney, 2018). Tau-U is a non-parametric effect size that combines non-overlap between phases with an adjustment for baseline trend. This metric is particularly suitable for SCED data as it is robust to non-normality and small sample sizes, can control for undesirable baseline trends, and provides an effect estimate that can be intuitively interpreted on a scale from −1 to +1 (Lee and Cherney, 2018; Shadish, 2014). Its use is increasingly recommended in SCED meta-analyses to supplement visual analysis and provide a standardized measure of effect (Kratochwill et al., 2021; Vannest et al., 2018).

Building upon the identified gaps, the present study has two primary objectives, operationalized through the following research questions:

(1) Overall Effectiveness: What is the aggregated effect size (Tau-U) of Social Story interventions on improving target behaviors and skills in individuals with ASD, based on the synthesis of single-case experimental studies?

(2) Moderating Effects: Does the magnitude of the intervention effect vary significantly as a function of key participant and intervention characteristics? Specifically, we will examine the potential moderating roles of:

Participant Age (e.g., preschool vs. school-age).

Intervention Target (e.g., social skills, problem behavior, adaptive/self-care skills).

Story Format (digital vs. paper-based).

Implementer Identity (therapist, teacher, parent, researcher).

Participant Gender.

By addressing these questions, this meta-analysis aims to move beyond the question of whether Social Stories work on average, toward a more nuanced understanding of the conditions under which they are most effective. The findings are expected to inform more targeted and individualized application in both clinical and educational practice, and to guide future research by highlighting critical factors that influence intervention outcomes.

## 2 Methods

### 2.1 Literature search strategy

A systematic search was conducted to identify all relevant single-case experimental design (SCED) studies on Social Story interventions for individuals with ASD. The five major databases (Web of Science, PubMed (MEDLINE), Embase, SpringerLink, and Academic Search Premier (ASP)) were selected because they collectively index the major peer-reviewed literature in psychology, education, special education, and biomedical sciences, ensuring comprehensive coverage of Social Story intervention studies for ASD. The five electronic databases were searched from their inception up to January 2026. The search strategy employed a combination of subject headings (e.g., MeSH terms) and free-text keywords related to the population and intervention. The full search strategy for PubMed was: (“Autism Spectrum Disorder”[MeSH Terms] OR ASD OR Autis^*^) AND (“Social Stor^*^” OR “Social Narrative^*^” OR “Carol Gray”). Equivalent search strings were adapted for the other databases. To ensure comprehensiveness, the initial search was deliberately broad and did not include methodological filters for SCEDs; such studies were identified through subsequent manual screening of titles and abstracts (Wright et al., 2016). Although no language restrictions were applied during the initial search to maximize sensitivity, only English-language studies were included in the final analysis because the meta-analysis required consistent data extraction and effect size calculation from graphs and tables, which could not be reliably performed for non-English articles due to translation limitations and potential misinterpretation of methodological details. The search and reporting process followed the Preferred Reporting Items for Systematic Reviews and Meta-Analyses (PRISMA) guidelines (Zhou k. et al., 2024). The initial database searches yielded a total of 1,581 records.

### 2.2 Inclusion and exclusion criteria

Studies were included if they met all of the following criteria:

(1) Study Design: Employed a single-case experimental design (e.g., AB, ABA, reversal, multiple baseline across participants, behaviors, or settings) with clearly defined baseline and intervention phases (Wright et al., 2014; Kokina and Kern, 2010; Tate et al., 2013; Kratochwill and Levin, 2014).

(2) Participants: Included at least one participant with a formal diagnosis of Autism Spectrum Disorder (ASD) according to the Diagnostic and Statistical Manual of Mental Disorders (DSM-IV-TR, DSM-5) or the International Classification of Diseases (ICD-10). Age and gender were not restricted.

(3) Intervention: Implemented a Social Story intervention as the primary independent variable, with the story development reportedly adhering to the guidelines proposed by Gray (2010) (e.g., inclusion of descriptive, perspective, directive, and affirmative sentences) (Wright et al., 2014).

(4) Data Presentation: Provided clear graphical or tabular data for both baseline and intervention phases, allowing for the extraction of data points necessary for effect size calculation.

(5) Publication Type: Peer-reviewed journal articles published in English.

Studies were excluded if they were: (1) non-interventional (e.g., reviews, theoretical papers, qualitative studies); (2) did not use Social Story as the core intervention; (3) involved participants without an ASD diagnosis; (4) presented incomplete or irretrievable data; or (5) were dissertations, conference abstracts, or book chapters.

We did not exclude studies based on a quality threshold because excluding lower-quality studies would have substantially reduced the sample size and potentially introduced selection bias, while the RoBiNT scores of included studies were predominantly in the moderate range (median internal validity score = 10, range 7–13).

### 2.3 Study selection and PRISMA flow

**Figure 1:**
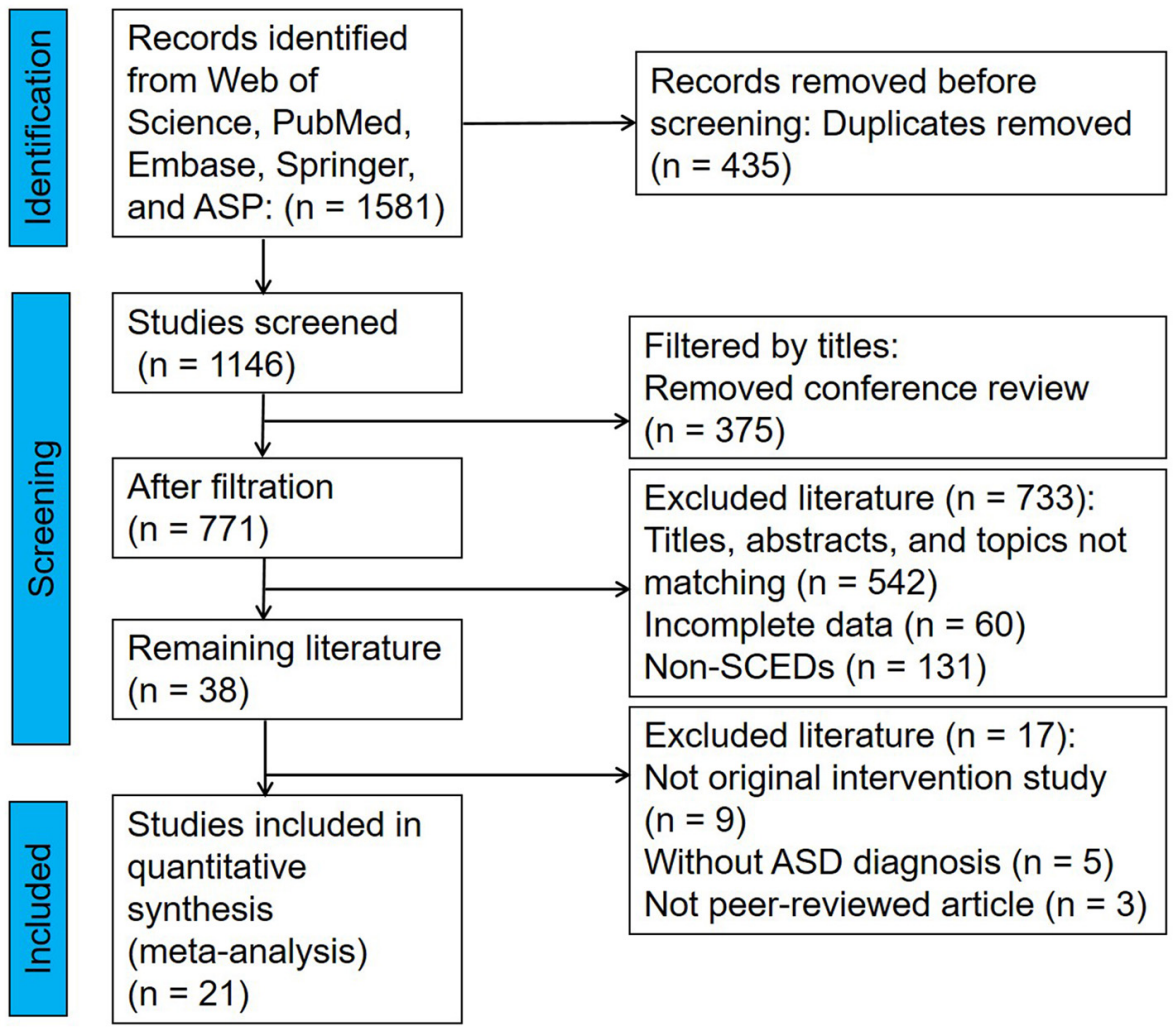
The PRISMA flow diagram illustrating the identification and screening of included studies.

The study selection process adhered to the PRISMA 2020 statement and flow diagram for reporting systematic reviews (Page et al., 2021a,b). After removing duplicates (*n* = 435), 1,581 records were screened by title and abstract. Of these, 375 were excluded as they are conference review. After that, 733 were excluded as they did not meet the inclusion criteria (e.g., 131 non-SCED designs, 60 incomplete data, 542 not matching). The remaining 38 full-text articles were assessed for eligibility. A further 17 full-text articles were excluded for the following reasons: not an original intervention study (e.g., review, *n* = 9), participants without ASD diagnosis (*n* = 5), or not a peer-reviewed journal article (*n* = 3). Consequently, 21 studies met all inclusion criteria and were retained for the meta-analysis. The titles and abstracts of the remaining records were screened independently by two reviewers against the inclusion criteria. Potentially relevant articles were retrieved for full-text review. The two reviewers then independently assessed the full-text articles for final eligibility. Simple percentage agreement was calculated as the number of items with identical ratings divided by the total number of items, multiplied by 100. In addition, Cohen's kappa was computed for the internal validity and external validity subscales, yielding κ = 0.79 and κ = 0.74, respectively, indicating substantial to excellent agreement. Inter-rater agreement for title/abstract screening was 92.4%, and for full-text eligibility was 96.7%. All disagreements at any stage were resolved through discussion or consultation with a third researcher. A summary of the screening process is presented in Figure 1.

### 2.4 Coding procedures and variables nomenclature

A standardized coding protocol was developed to extract relevant data from the included studies. Coding was performed independently by two researchers. The extracted variables included:

• Participant Characteristics: Number of participants, mean/range of age, gender distribution.

• Intervention Characteristics: Format of the Social Story (paper-based vs. digital), identity of the primary implementer (therapist, teacher, parent, researcher), intervention setting (school, clinic, home), and target behavior category. The selection of these intervention-related moderators was informed by prior meta-analytic work in this field (Kokina and Kern, 2010).

• Outcome Domains: Target behaviors were categorized into four primary domains for moderator analysis: Social Skills (e.g., initiations, responses), Problem Behavior (e.g., aggression, tantrums), Safety/Adaptive Skills (e.g., crossing street, toothbrushing), and Classroom Adaptive Skills (e.g., on-task behavior, following routines).

• Study Design and Quality: Design type and data for quality assessment. The methodological quality of each included single-case experimental design study was assessed using the Risk of Bias in N-of-1 Trials (RoBiNT) Scale (Tate et al., 2013). The RoBiNT Scale comprises two subscales: the Internal Validity Subscale (7 items) evaluates the risk of bias, and the External Validity/Interpretability Subscale (8 items) evaluates the quality and generalizability of the findings. Each item was rated as “Yes” (2 points), “Partially” (1 point), or “No” (0 point). Two reviewers independently conducted the ratings, with any discrepancies resolved through discussion or consultation with a third researcher. Inter-rater agreement prior to consensus exceeded 85% for both subscales. The detailed scoring criteria for each item are presented in Table 1 (Internal Validity) and Table 2 (External Validity and Interpretation). The results of this quality assessment are reported in the Results section (Table 3).

**Table 1:** Internal validity.

Item No.	Evaluation content	Scoring criteria and key explanations
IV.1	**Participant and baseline characteristics:** Were relevant participant characteristics (e.g., demographics, diagnosis, functional status) sufficiently described?	**Yes:** Detailed description, with characteristics clearly relevant to the intervention target. **Partially:** Incomplete description. **No:** Not described or description is entirely irrelevant.
IV.2	**Dependent variable:** Was the dependent variable (target outcome) quantified and measured using objective or validated methods?	**Yes:** Completely objective (e.g., frequency of correct responses) or used tools with published validity evidence (e.g., standardized scales). **Partially:** Combined objective and subjective methods. **No:** Relied entirely on subjective evaluation.
IV.3	**Independent variable:** Was the independent variable (intervention) operationally defined and described with sufficient clarity to permit precise replication?	**Yes:** Provided step-by-step, specific operational procedures. **Partially:** Described general procedures but lacked key details. **No:** Vague description, precluding replication.
IV.4	**Baseline phase:** Were baseline data presented for a sufficient duration prior to intervention, with a stable pattern (or a trend in the opposite direction of the expected intervention effect)?	**Yes:** ≥3 baseline data points with a stable trend or a trend in the undesired direction. **Partially:** Sufficient data points but with an unstable trend in the same direction as the intervention effect. **No:** Baseline phase too short (<3 points) or showed a clear improving trend in the same direction as the intervention.
IV.5	**Blinding:** Was blinding implemented for assessors (or participants, if feasible)?	**Yes:** Explicitly reported effective blinding of outcome assessors (or participants). **Partially:** Partial blinding or methods with flaws. **No:** Blinding not used or not mentioned.
IV.6	**Randomization:** Was the sequence of interventions or the timing of phase changes determined by a randomization procedure?	**Yes:** Explicitly reported using a randomization procedure (e.g., random number table) to determine the intervention sequence or phase change points. **Partially:** Used non-random, systematic alternation (e.g., rotation). **No:** Used a non-random, subjectively determined method.
IV.7	**Data reporting:** Were all data points for all phases and all participants reported? (For graphical data, all data points must be included).	**Yes:** All data points included in graphs or tables, with no omissions. **Partially:** Reported aggregated data (e.g., means), or had a few missing points with reasonable explanation. **No:** Evidence of selective reporting or substantial missing data.

**Table 2:** External validity and interpretation.

Item no.	Evaluation content	Scoring criteria and key explanations
EV/Int.8	**Design appropriateness:** Did the research design allow for at least three demonstrations of intervention effect at different points in time?	**Yes:** The design inherently included at least three effect demonstrations (e.g., A-B-A-B design has 3 phase changes; multiple-baseline design across 3 participants). **Partially:** Provided two demonstrations. **No:** Only one demonstration (e.g., A-B design).
EV/Int.9	**Effect replicability:** Was the intervention effect clearly demonstrated across time, across participants, behaviors, or settings?	**Yes:** Effects were clear and consistent across all preset replication units. **Partially:** Clear in some, but not all, units. **No:** Effects were inconsistent or ambiguous.
EV/Int.10	**Effect contiguity:** Did changes in the dependent variable correspond closely in time to the manipulation of the independent variable?	**Yes:** Changes in the dependent variable immediately followed the introduction or withdrawal of the independent variable. **Partially:** Changes were delayed but clearly associated with the intervention. **No:** No temporal relationship between changes and intervention manipulation.
EV/Int.11	**Data Analysis:** Were statistical methods or effect size calculations used to analyze the data?	**Yes:** Used appropriate statistical tests or effect size metrics (e.g., non-overlap indices, regression models, randomization tests). **Partially:** Only descriptive summaries of data (e.g., mean comparisons). **No:** No quantitative analysis was used.
EV/Int.12	**Effect magnitude and significance:** Did the study report the magnitude of the intervention effect and discuss its clinical/practical significance?	**Yes:** Reported effect size and interpreted its practical importance. **Partially:** Only described changes without quantification, or quantified without discussing significance. **No:** Not addressed.
EV/Int.13	**Measurement reliability:** Was the reliability of dependent variable measurement assessed and reported?	**Yes:** Systematically assessed and reported acceptable reliability indices (e.g., ICC > 0.75, agreement > 80%). **Partially:** Assessed non-systematically or reliability at borderline levels. **No:** Not assessed or reported.
EV/Int.14	**Procedural fidelity:** Was the fidelity of intervention implementation assessed and reported?	**Yes:** Systematically assessed and reported high implementation fidelity (e.g., > 80%). **Partially:** Assessed non-systematically or fidelity was moderate. **No:** Not assessed or reported.
EV/Int.15	**Social validity:** Was the social importance/acceptability of intervention goals, procedures, or outcomes assessed?	**Yes:** Formally assessed social validity using methods such as questionnaires or interviews. **Partially:** Only included informal comments. **No:** Not addressed.

**Table 3:** Results of the methodological quality assessment.

No.	Author (year)	Internal validity score (risk of bias)	External validity and interpretation score (quality of interpretation)
1	Kurt and Kutlu (2019)	10 (moderate risk)	16 (high quality)
2	Ulaşmanand Sivrikaya (2025)	10 (moderate risk)	16 (high quality)
3	Klett and Turan (2012)	9 (moderate risk)	9 (moderate quality)
4	Chan et al. (2011)	10 (moderate risk)	12 (moderate quality)
5	O'Handley et al. (2015)	13 (low risk)	14 (moderate-high quality)
6	Halle et al. (2016)	11 (low risk)	13 (moderate-high quality)
7	Daneshvar et al. (2018)	10 (moderate risk)	10 (moderate quality)
8	Anthony and Bobzien (2022)	10 (moderate risk)	13 (moderate quality)
9	Samuels and Stansfield (2011)	9 (moderate risk)	6 (low quality)
10	Scattone et al. (2006)	10 (moderate risk)	15 (good quality)
11	Thiemann and Goldstein (2001)	10 (moderate risk)	14 (good quality)
12	Sansosti and Powell-Smith (2008)	10 (moderate risk)	14 (good quality)
13	Kuoch and Mirenda (2003)	8 (moderate risk)	10 (moderate quality)
14	Ozdemir (2008)	9 (moderate risk)	11 (moderate-high quality)
15	Lorimer et al. (2002)	7 (moderate risk)	9 (moderate quality)
16	Scattone et al. (2002)	10 (moderate risk)	13 (moderate quality)
17	Vandermeer et al. (2015)	9 (moderate risk)	13 (moderate quality)
18	Mancil et al. (2009)	10 (moderate risk)	14 (moderate quality)
19	Xue et al. (2025)	11 (low risk)	14 (high quality)
20	Kwon et al. (2023)	9 (moderate risk)	11 (moderate quality)
21	Lee and Hsu (2024)	8 (moderate risk)	13 (high quality)

Intercoder agreement for all other categorical and continuous variables (e.g., participant demographics, intervention characteristics) was calculated prior to consensus discussions. Initial agreement exceeded 90% for all major coding categories.

### 2.5 Effect size calculation and data analysis

#### 2.5.1 Effect size metric

The Tau-U nonparametric effect size was selected as the primary index (Lee and Cherney, 2018; Parker et al., 2011). Tau-U combines non-overlap between phases with an adjustment for baseline trend, making it particularly suitable for SCED data (Lee and Cherney, 2018; Parker et al., 2011). It produces an effect estimate ranging from −1 to +1, where positive values indicate improvement. Tau-U was calculated for each phase contrast (baseline vs. intervention) within each participant and outcome using the online calculator developed by Parker and Vannest (http://www.singlecaseresearch.org). For studies targeting the reduction of problem behaviors, the negative effect size was reversed to a positive value to allow for meaningful aggregation of effects across studies. Consistent with established benchmarks, effect sizes were interpreted as: negligible (<0.48), small (0.48–0.65), medium (0.66–0.81), large (0.82–0.92), and very large (>0.93) (Parker et al., 2011; Guzman et al., 2018; Aydin et al., 2025).

#### 2.5.2 Data extraction and reliability

Data points were extracted from published graphs using digital extraction software. Two researchers independently extracted data and calculated Tau-U effects. The final inter-rater reliability for effect size calculation was 92.24%. The 92.24% inter-rater reliability for effect size calculation was computed as the proportion of Tau-U values that were within ±0.05 between the two raters, divided by the total number of calculated effect sizes. Discrepancies beyond this threshold were resolved by joint re-examination.

#### 2.5.3 Aggregation and moderator analysis

An overall weighted mean Tau-U was calculated across all participants and studies (Guzman et al., 2018; Aydin et al., 2025; Zhou k. et al., 2024). To address the nested structure of the data (multiple effects within studies), appropriate meta-analytic models were employed. Planned moderator analyses were conducted to examine whether the effect size varied systematically as a function of: participant age group, target behavior domain, Social Story format (digital vs. paper), and implementer identity (Aydin et al., 2025; Zhou k. et al., 2024). All analyses were conducted using R software with the “metafor” package.

## 3 Results

### 3.1 Characteristics of included studies conflict of interest

A total of 21 single-case experimental design studies, published between 2001 and 2025, were included in the meta-analysis, encompassing data from 61 participants with ASD. The methodological quality of the included studies, assessed using the RoBiNT scale, was acceptable overall. In terms of internal validity (risk of bias), the majority of studies (*n* = 18, 85.7%) were rated as having a moderate risk of bias, while three studies (14.3%) were rated as low risk. Regarding external validity/interpretability, over half of the studies (*n* = 11, 52.4%) demonstrated good to high interpretability. To examine the potential impact of study quality on our findings, we conducted a sensitivity analysis by excluding the three studies with the lowest internal validity scores (RoBiNT ≤ 8) and recalculated the overall effect size. The Tau-U remained 0.74 [95% CI (0.68, 0.80)], indicating that the overall conclusion was robust.

Participant characteristics are summarized in Table 4. The age of participants ranged from 3 to 32 years, with school-aged children (7–12 years) constituting the largest subgroup. The sample was predominantly male (*n* = 55), with only six female participants, reflecting the common gender distribution in ASD research. In terms of intervention characteristics, digital Social Stories (*n* = 12 studies) were slightly more common than paper-based formats (*n* = 9 studies). The primary target behaviors were categorized as Social Skills (*n* = 9 studies), Problem Behavior (*n* = 8 studies), Safety Skills (*n* = 3 studies), and Classroom Adaptive Skills (*n* = 1 study). Interventions were most frequently conducted in classroom (*n* = 11) or therapy room (*n* = 7) settings and were implemented by a range of agents, including teachers (*n* = 9), therapists (*n* = 6), researchers (*n* = 5), and parents (*n* = 3). The detailed characteristics of each included study are summarized in Table 4. The methodological quality of the included studies, assessed using the RoBiNT scale (see Methods, Section 2.4 for criteria), was accepted overall. The detailed ratings for each study are presented in Table 3.

**Table 4:** Characteristics of included studies.

No.	Author (year)	Age (years)	Gender (F;M)	Intervention format	Target behavior/skill	Setting	Intervention agent
1	Kurt and Kutlu (2019)	4–12	0; 3	Digital Social Story	Safety skills	Therapy room	Therapist
2	Ulaşmanand Sivrikaya (2025)	10–11	2; 1	Digital Social Story	Safety skills	Classroom	Teacher
3	Klett and Turan (2012)	9–12	3; 0	Paper Social Story	Safety skills	Home	Parent
4	Chan et al. (2011)	8	0; 3	Paper Social Story	Classroom adaptive skills	Classroom	Teacher
5	O'Handley et al. (2015)	18	0; 1	Digital Social Story	Social skills	Classroom	Researcher
6	Halle et al. (2016)	12–14	1; 3	Digital Social Story	Social skills	Classroom	Teacher
7	Daneshvar et al. (2018)	6–11	1; 3	Digital Social Story	Social skills	Therapy room	Researcher
8	Anthony and Bobzien (2022)	13	0; 2	Digital Social Story	Social skills	Therapy room	Therapist
9	Samuels and Stansfield (2011)	20–32	0; 3	Paper Social Story	Social skills	Home	Therapist
10	Scattone, Tingstrom, and Wilczynski (2006)	8–13	0; 3	Paper Social Story	Social skills	Classroom	Teacher
11	Thiemann and Goldstein (2001)	6–11	0; 4	Paper Social Story	Social skills	Classroom	Researcher
12	Sansosti and Powell-Smith (2008)	6–9	0; 3	Digital Social Story	Social skills	Classroom	Teacher
13	Kuoch and Mirenda (2003)	3–6	0; 3	Paper Social Story	Problem behavior	Classroom	Parent
14	Ozdemir (2008)	7–9	0; 3	Paper Social Story	Problem behavior	Classroom	Teacher
15	Lorimer et al. (2002)	5	0; 1	Paper Social Story	Problem behavior	Home	Parent
16	Scattone et al. (2002)	7–15	0; 3	Paper Social Story	Problem behavior	Classroom	Teacher
17	Vandermeer et al. (2015)	4	1; 2	Digital Social Story	Problem behavior	Classroom	Researcher
18	Mancil et al. (2009)	6–8	1; 2	Digital Social Story	Problem behavior	Classroom	Teacher
19	Xue et al. (2025)	10	0; 2	Digital Social Story	Problem behavior	Therapy Room	Therapist
20	Kwon et al. (2023)	7–10	0; 3	Paper Social Story	Problem behavior	Classroom	Researcher
21	Lee and Hsu (2024)	6–9	0; 4	Digital Social Story	Social skills	Therapy room	Therapist

### 3.2 Overall effectiveness of social story interventions

The aggregated effect size across all 61 participants and 21 studies was Tau-U = 0.743 [90% CI (0.682, 0.804), *p* < 0.001]. The heterogeneity test indicated no significant variability in effects across studies (Q = 46.78, *p* = 0.8937; *I*^2^ = 0.0%). Although the overall *I*^2^ was 0%, we proceeded with planned moderator analyses because theoretical considerations and previous meta-analyses suggest that participant and intervention characteristics may still meaningfully influence outcomes, and examining moderators can reveal nuanced patterns not captured by a single aggregated effect. According to established benchmarks for Tau-U, this represents a moderate overall effect size, indicating that Social Story interventions are, on average, an effective approach for improving target behaviors and skills in individuals with ASD.

### 3.3 Moderator analyses

To examine potential sources of variability in effect sizes, moderator analyses were conducted for key participant and intervention characteristics.

#### 3.3.1 Age group

The intervention effect varied across age groups. The strongest effects were observed for school-aged children (7–12 years), with a Tau-U of 0.63 [95% CI (0.55, 0.71)]. Preschool children (3–6 years), adolescents (13–18 years), and adults (>18 years) also showed positive effects (Tau-U = 0.59, 0.58, and 0.55, respectively), though the confidence intervals were wider for the adolescent and adult groups, likely due to smaller sample sizes. The test for differences between these age subgroups was not statistically significant (Q_between = 0.45, *p* = 0.930).

#### 3.3.2 Target behavior domain

Significant positive effects were found for interventions targeting Social Skills [Tau-U = 0.59, 95% CI (0.50, 0.68)], Safety Skills [Tau-U = 0.66, 95% CI (0.47, 0.85)], and Problem Behavior (Tau-U = 0.63, 95% CI [0.52, 0.74]). In contrast, the effect for Classroom Adaptive Skills was non-significant and smaller in magnitude [Tau-U = 0.40, 95% CI (0.02, 0.78)], suggesting Social Stories may be less effective for this specific domain based on the limited available evidence. The differences across behavior domains were statistically significant (Q_betwee*n* = 1.69, *p* = 0.640).

#### 3.3.3 Intervention format

A notable difference was found based on the format of the Social Story. Digital Social Stories yielded a higher average effect size [Tau-U = 0.64, 95% CI (0.54, 0.73)] compared to Paper-based Social Stories [Tau-U = 0.58, 95% CI (0.49, 0.67)]. However, the analysis indicated that this difference was not statistically significant (Q_betwee*n* = 0.68, *p* = 0.410).

#### 3.3.4 Implementer Type and Gender

The identity of the intervention implementer—whether therapist, teacher, parent, or researcher—did not significantly moderate the intervention effect. All groups produced significant positive effects, with point estimates ranging from Tau-U = 0.51 (researchers) to 0.65 (therapists). Differences between implementer types were not significant (Q_betwee*n* = 2.89, *p* = 0.409). Similarly, while the sample was disproportionately male, analysis indicated that both males (Tau-U = 0.62) and females (Tau-U = 0.51) benefited from the intervention, with no significant moderating effect of gender detected (Q_betwee*n* = 1.31, *p* = 0.252).

**Figure 2:**
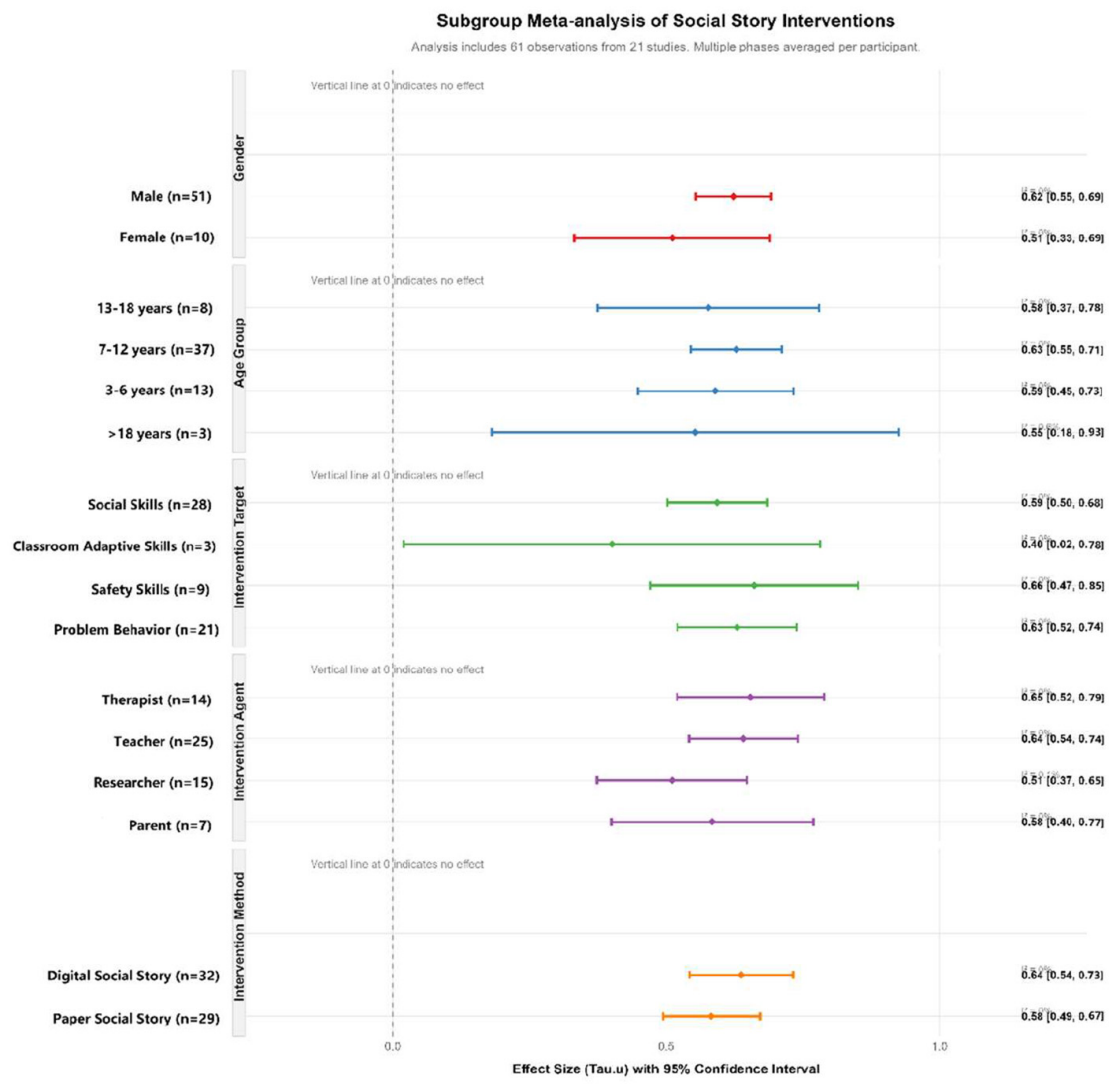
Summary of forest plots of moderator analysis for Social Story intervention effects.

The forest plots for all moderator analyses are summarized in Figure 2.

### 3.4 Publication bias

**Figure 3:**
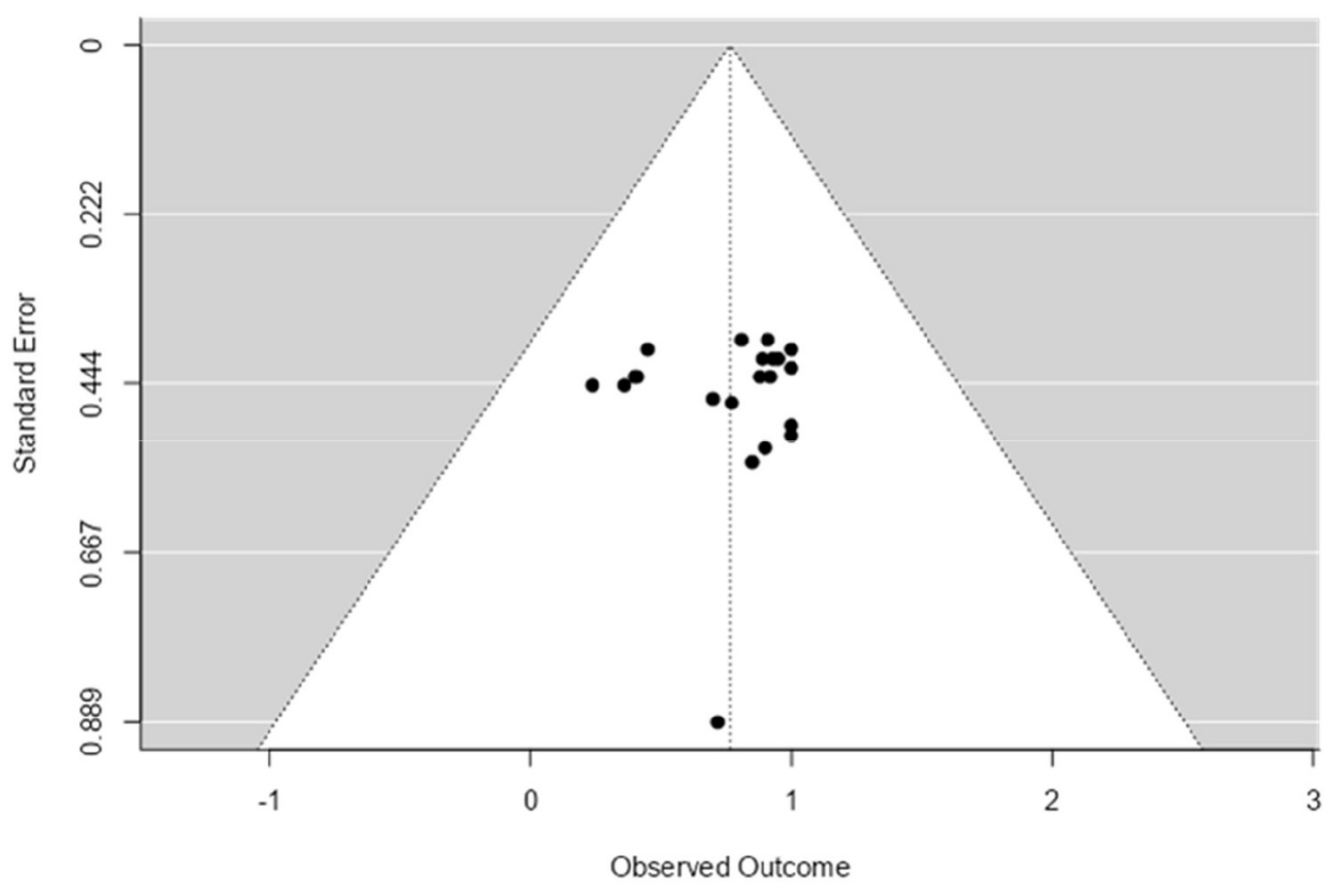
Funnel plot of included studies: effect size and standard error.

Visual inspection of the funnel plot (Figure 3) showed a roughly symmetric distribution of effect sizes around the overall mean, suggesting a low risk of publication bias. This was supported by Egger's regression test (*t* = 0.301, *p* = 0.765).

## 4 Discussion

This meta-analysis synthesized data from 21 single-case experimental design studies, encompassing 61 participants with ASD, to evaluate the overall effectiveness of Social Story interventions and investigate key moderating factors. The findings contribute to the evolving evidence base by providing a quantitative synthesis that both confirms and refines our understanding of this widely used practice.

### 4.1 Principal findings and overall efficacy

This meta-analysis reveals a moderate overall effect (Tau-U = 0.743) of Social Story interventions for individuals with ASD, confirming their efficacy as an evidence-informed approach. This finding aligns with a recent synthesis (Tang et al., 2023) but provides a crucial update to earlier conclusions that characterized the intervention's effectiveness as “low to questionable” (Kokina and Kern, 2010). Additionally, we performed a meta-regression with internal validity score as a continuous moderator; the effect was not significant (p > 0.05), suggesting that study quality did not systematically influence the observed effect sizes. The more robust effect observed here likely reflects key methodological advancements: (1) the use of the Tau-U metric, which adjusts for baseline trend and offers a more precise estimate than earlier non-overlap indices; (2) the inclusion of more recent studies (up to 2025) that potentially demonstrate improved procedural fidelity and design rigor; and (3) the explanatory model incorporating significant moderators (e.g., age, target behavior), which moves beyond a single heterogeneous estimate to clarify when and for whom the intervention works best. Consequently, our results affirm that Social Stories can yield meaningful effects when thoughtfully individualized, reinforcing their value in practice. The significant heterogeneity quantified in our analysis further underscores the necessity of this moderator-driven perspective.

Tang et al. (2023) conducted a meta-analysis by using articles published between 2010 and 2022, however, our search extended to January 2026, including 4 new studies published after 2022 that were not covered by Tang et al. We systematically examined a wider range of moderators (age, target behavior, format, implementer, gender) and reported non-significant findings (e.g., digital vs. paper, implementer type), which were not explored in the previous review. We conducted sensitivity analyses to assess the impact of study quality, whereas Tang et al. (2023) did not report such analyses.

### 4.2 Interpretations and contextualization of key findings

#### 4.2.1 Age and target behavior specificity

Our moderator analysis revealed that Social Story interventions were most effective for school-aged children (7–12 years), while yielding a non-significant effect for Classroom Adaptive Skills (e.g., on-task behavior). This pattern can be meaningfully interpreted through the lens of intervention mechanism and target behavior complexity.

The peak efficacy in middle childhood likely coincides with a critical developmental window where children have acquired the necessary receptive language and symbolic understanding to decode narrative-based instructions, yet remain highly receptive to learning explicit social scripts. This aligns with findings from computer-based social skills interventions, which are also effectively deployed in this age group to teach facial emotion recognition and mentalizing in a structured, predictable manner (Ramdoss et al., 2012). Both Social Stories and such computer-based training share a common strength: they break down complex social information into discrete, rule-based components, a format that may be particularly accessible to school-aged children with ASD (Lu et al., 2019).

Conversely, the diminished effect on Classroom Adaptive Skills suggests a boundary condition for Social Stories. Behaviors like sustained attention and routine-following are less about misunderstanding a specific social cue and more dependent on core executive functions (e.g., inhibitory control, cognitive flexibility) and broad behavioral regulation. A meta-analysis of group social skills interventions similarly found that outcomes vary significantly based on participant characteristics, with co-occurring conditions like ADHD influencing treatment response (Gerber et al., 2023). It is plausible that the challenges underlying classroom adaptation often involve such broader, non-social cognitive processes, which are not the primary target of a social narrative intervention. This distinction echoes earlier findings that Social Stories are more effective for reducing clearly defined “inappropriate behaviors” than for teaching broad social skills (Kokina and Kern, 2010).

Furthermore, the dynamic, complex, and less predictable nature of the classroom environment itself may be a factor. While Social Stories excel at preparing an individual for a specific situation, generalizing that learning to the fluid and multi-demand context of a classroom is a greater challenge. This underscores the importance of ecological validity in intervention design—a principle highlighted in discussions of advanced technologies like virtual reality, which aim to train skills in simulated yet realistic environments (Banire et al., 2020). The non-significant finding for classroom skills signals a need either to integrate Social Stories within more comprehensive classroom support frameworks or to explore enhanced story formats that better address generalization to complex, real-world settings.

#### 4.2.2 The nuanced role of digital delivery

A pivotal finding of this meta-analysis is the nuanced role of digital delivery. While digital Social Stories demonstrated a higher point estimate of effect (Tau-U = 0.64) compared to paper-based formats (Tau-U = 0.58), this difference was not statistically significant (*p* = 0.410). This result offers a critical, evidence-based perspective on the technological enhancement of traditional interventions.

The positive trend aligns with the proposed advantages of digital mediation. Digital platforms can standardize delivery, reduce implementation variability, and incorporate multimedia elements (e.g., audio narration, animations) that may enhance engagement and comprehension for individuals with ASD (Hanrahan et al., 2020; Camilleri et al., 2022). Research indicates that digitally-mediated Social Stories can effectively produce beneficial behavioral changes (Hanrahan et al., 2020) and are perceived by parents and practitioners as tools that improve their own competence and intervention fidelity (Camilleri et al., 2022). This suggests that the digital format may act as a potent facilitator by increasing the accessibility, consistency, and perceived usability of the intervention.

However, the lack of statistical significance is a crucial empirical observation. It implies that the core therapeutic ingredient of Social Stories may reside in the structured, individualized social narrative itself—as defined by Gray's criteria—rather than solely in the medium of delivery. The digital format, despite its practical and logistical benefits, may not inherently amplify the intervention's primary psychological mechanism of providing predictable social information. This interpretation is supported by a broader meta-analysis of digital interventions for ASD, which found significant positive effects but also highlighted substantial variability across studies (Xu et al., 2026). The category “digital” in our analysis encompassed a heterogeneous mix, from static PDFs viewed on tablets to interactive applications. This broad definition likely diluted specific effects, as the level of interactivity and personalization—key features in serious games or embodied conversational agents that show efficacy in social skills training (Talebi Azadboni et al., 2024; Tanaka et al., 2017) —varies widely. Thus, the non-significant difference may reflect the current conceptual and methodological heterogeneity within “digital” Social Story research, rather than a definitive absence of effect.

Therefore, future research must move beyond a simple binary comparison of “digital vs. paper”. Instead, the focus should shift to investigating which specific digital features (e.g., interactive choice-making, personalized avatars, embedded performance feedback, or simulation of social contexts (Lu et al., 2019; Banire et al., 2020) may interact with the Social Story framework to create additive or synergistic therapeutic value. This requires carefully designed, component-based studies that distinguish the logistical advantages of digital platforms from any genuine enhancement of the intervention's core change mechanism.

#### 4.2.3 Implementer flexibility and generalizability

Our finding that intervention effectiveness was not significantly moderated by implementer identity (therapist, teacher, parent, researcher) is highly encouraging for the practical dissemination and ecological validity of Social Stories. It suggests that the core protocol—a structured, individualized narrative—is sufficiently robust to be delivered effectively by a variety of key agents within the individual's natural environment, provided they are adequately guided in its use. This flexibility is a significant strength, as it enhances the intervention's scalability and potential for integration into daily routines at school and home. Feasibility studies have successfully demonstrated the implementation of Social Stories by teachers in mainstream classrooms (Marshall et al., 2016), and exploratory research supports the potential for digitally-mediated, parent-led delivery (Camilleri et al., 2024).

This finding invites a nuanced discussion when contrasted with prior research. An earlier meta-analysis suggested that implementation by natural agents (e.g., teachers) or the target children themselves was associated with more pronounced effects (Kokina and Kern, 2010). The discrepancy with our results may be attributed to the evolving research landscape. Earlier studies might have reflected a wider variability in how non-specialist implementers were trained and supported, whereas more recent studies included in our analysis may involve better-standardized protocols or training materials, reducing performance disparities between professional and non-professional implementers. Furthermore, the rise of digital tools may be a key mediating factor, as they can provide structured support that enhances implementation fidelity across different user groups (Camilleri et al., 2022, 2024).

Therefore, a balanced interpretation is that while a range of individuals can be effective implementers, success is likely contingent on adequate preparation and support. The critical factor may not be the implementer's formal role, but rather their access to clear guidelines, appropriate resources (potentially digital), and an understanding of how to personalize and present the story effectively. This shifts the practical implication from simply identifying the “best” implementer to ensuring that any implementer is well-equipped, thereby maximizing the intervention's reach and real-world impact.

### 4.3 Methodological limitations and their implications

The conclusions of this meta-analysis must be interpreted within the context of its limitations, which primarily reflect the current state of the primary research literature.

#### 4.3.1 Limitations inherent to the evidence base

The strength and generalizability of our conclusions are constrained by several characteristics common to the existing body of Social Story research. First, the evidence base is dominated by single-case experimental designs (SCEDs). While SCEDs provide high internal validity for demonstrating functional relationships at the individual level, their aggregated results, including ours, must be generalized with caution to the broader ASD population (Kokina and Kern, 2010). This design dominance is a recognized feature of the field, as noted in prior syntheses (Kokina and Kern, 2010).

Second, there is a pronounced lack of demographic diversity within the included studies. Participants were overwhelmingly male, of school age, and likely represent a subset of the spectrum with fewer co-occurring intellectual disabilities. This reflects a wider research bias in ASD intervention studies and severely limits our ability to ascertain whether Social Stories are equally effective for adolescents, adults, females, and individuals with greater support needs (Kokina and Kern, 2010). The efficacy observed here may not be fully representative of the intervention's impact across the full heterogeneity of ASD.

Third, consistent with previous reviews, the primary studies exhibited a near-universal absence of long-term follow-up data (Thiemann and Goldstein, 2001; Zhang et al., 2021). Most studies measured outcomes only immediately post-intervention, leaving a critical gap in our understanding of the maintenance and generalization of skills over time and across settings. Consequently, our meta-analysis can speak to the short-term, context-specific efficacy of Social Stories but cannot address their value in producing lasting, generalized behavioral change. This remains a key priority for future high-quality research.

#### 4.3.2 Limitations of the present meta-analysis

Several methodological limitations specific to this review warrant consideration. First, our analysis was constrained by the detail of reporting in the primary studies. Consequently, several important potential moderators that could influence effectiveness—such as intervention intensity (frequency, duration), quantitative measures of implementation fidelity, baseline participant language level, and the specific compositional quality of the stories (e.g., adherence to Gray's sentence ratio guidelines)—could not be systematically examined. The influence of factors like language level and implementation support has been noted in related digital intervention research (Camilleri et al., 2024).

Second, and most critically for interpreting a key finding, the analysis of digital delivery format has two primary limitations. The categorization “digital” encompassed a heterogeneous range of technologies, from static PDFs viewed on a screen to interactive applications with built-in prompts. This broad definition, while necessary due to reporting practices, likely masked potential differential effects, as the level of interactivity and personalization is a known source of variability in digital intervention outcomes (Xu et al., 2026). More importantly, the statistical power of this subgroup comparison may have been limited. The non-significant result (*p* = 0.410) should therefore be interpreted not as definitive evidence of “no difference”, but as an indication that any true difference between digital and paper-based formats, if it exists, may be small or detectable only with a larger, more homogenous sample of studies. This directly tempers the conclusions drawn in section 4.2.2.

Finally, although inter-rater agreement was high, the coding of variables (e.g., target behavior domain, implementer identity) and quality assessment involved a degree of subjective judgment, which is an inherent limitation of the meta-analytic process.

### 4.4 Practical implications for diverse settings

The findings of this meta-analysis translate into several concrete recommendations for practitioners across educational, clinical, and home settings. To maximize effectiveness, intervention planning must be highly individualized. Social Stories appear to be a particularly well-matched tool for school-aged children (7–12 years), especially when targeting goals related to understanding specific social cues, learning safety rules, or replacing a clearly defined problem behavior. For adolescents, adults, or goals centered on broad classroom compliance, practitioners should consider Social Stories as one component within a broader support strategy, managing expectations accordingly and potentially combining them with other evidence-based approaches.

Regarding format, digital Social Stories can be adopted pragmatically. Our findings, along with broader evidence supporting digital interventions (Xu et al., 2026), suggest they are at least as effective as traditional paper-based stories. Therefore, the choice of medium can be driven by practical considerations: digital formats offer advantages in ease of sharing, ensuring consistent presentation, and increasing engagement, particularly for tech-oriented learners. Developers and practitioners should focus on leveraging these strengths—such as incorporating interactive elements or personalized avatars based on learner preference—to enhance usability and adherence, rather than assuming technological complexity alone will yield superior outcomes.

Finally, the robust effect across implementers supports the active empowerment of natural support agents. Teachers can effectively integrate stories into the classroom routine to prevent or address social-behavioral challenges (Marshall et al., 2016), while parents can be trained to develop and deliver stories at home, especially with the aid of digital tools that support implementation fidelity (Camilleri et al., 2024). This cross-setting consistency, facilitated by a shared, understandable tool like a Social Story, is crucial for promoting generalization. Ultimately, successful implementation relies less on the professional title of the implementer and more on providing all caregivers with clear guidelines, adequate resources, and a focus on individualizing the story to the learner's specific context and needs.

This is a short text to acknowledge the contributions of specific colleagues, institutions, or agencies that aided the efforts of the authors.

### 4.5 Future research directions

To advance the field beyond the current evidence base and address the limitations identified, future research should pursue the following concrete pathways:

First, there is a pressing need for well-designed, head-to-head comparative trials. Specifically, adequately powered Randomized Controlled Trials (RCTs) should directly compare standardized digital and paper-based Social Story protocols while meticulously controlling for story content, implementation fidelity, and implementer training. Such studies would definitively answer whether digital delivery offers a true superiority in efficacy, or if its primary advantages are in cost-effectiveness, accessibility, and implementation consistency—a crucial distinction for resource allocation.

Second, research must move beyond simple digitization (e.g., PDFs on a tablet) to explore the integration of interactive and immersive technologies. Future studies should investigate whether embedding interactive elements (e.g., choice-making branches, personalized avatars, or immediate performance feedback) within digital Social Stories can enhance engagement and skill acquisition. More ambitiously, the field should explore how Virtual Reality (VR) and Augmented Reality (AR) could create safe, controlled, yet ecologically valid environments for practicing the skills outlined in a Social Story. For instance, a story about navigating a busy cafeteria could be practiced within a simulated VR environment, allowing for repeated, stress-free rehearsal (Zhao et al., 2022; Mittal et al., 2024). Emerging meta-analytic evidence supports the positive impact of immersive VR on the social and emotional skills of individuals with ASD (Mittal et al., 2024; Yang et al., 2025), suggesting strong potential for synergistic integration with narrative-based interventions like Social Stories.

Third, expanding participant diversity is non-negotiable. Studies must intentionally recruit underrepresented groups, including adolescents, adults, females, and minimally verbal individuals or those with co-occurring intellectual disabilities. This will clarify the boundaries of the intervention's effectiveness and guide truly individualized practice. Relatedly, a major gap that must be filled is the systematic investigation of long-term maintenance and generalization. Future RCTs must incorporate extended follow-up periods (e.g., 6–12 months) and measure generalization across settings, people, and related behaviors to determine if initial gains translate into lasting, functional improvement.

Finally, to optimize efficiency, research should begin to deconstruct the intervention's “active ingredients”. Component-analysis studies could examine which elements are essential (e.g., the specific sentence ratio, the inclusion of child-specific photographs, the level of child involvement in creation) and which are optional. This would allow for the development of more streamlined, potent, and personalized versions of the intervention.

## 5 Conclusion

This meta-analysis affirms that Social Story interventions produce a moderate overall positive effect for individuals with ASD, solidifying their status as a valuable evidence-informed practice. Crucially, the effect is moderated by age and target behavior, highlighting the importance of thoughtful individualization. The equivalent performance of digital and traditional formats offers practitioners flexible, evidence-based choices for implementation. By addressing the identified limitations through more diverse, rigorous, and component-focused research, the field can refine Social Story theory and practice to better serve the heterogeneous needs of the autism community.

### 5.1 Limitations

One limitation in this meta-analysis is the small number of studies that met our inclusion criteria. Additionally, there was significant heterogeneity among studies.

## Data Availability

The original contributions presented in the study are included in the article/supplementary material, further inquiries can be directed to the corresponding author.
